# Editorial: Trends of microbial technologies in rehabilitation of contaminated environments

**DOI:** 10.3389/fmicb.2023.1268002

**Published:** 2023-08-22

**Authors:** Hari Prasanna Deka Boruah, Puneet Singh Chauhan, Celin Acharya

**Affiliations:** ^1^Biotechnology Group, Government Model College, Kaziranga, Kaziranga, Assam, India; ^2^Microbial Technologies Division, CSIR-National Botanical Research Institute, Lucknow, India; ^3^Molecular Biology Division, Bhabha Atomic Research Centre, Mumbai, India

**Keywords:** contaminant, pollution, microbial remediation, microbial community, soil

## Introduction

Civilization coupled with the industrial revolution and population growth accelerates the degradation and contamination of the environment to a large extent. The contamination of the environment, including soil, water, and air, arises due to a myriad of human activities, such as industrialization, modern agricultural practices, the release of unwanted chemicals, minerals, and effluents released from different industries and municipalities, and the dumping of waste materials and gas. Consequently, the contaminated environment has become a threat to the sustainable environment and is of serious concern. To contain and rehabilitate contaminated environments, microbial technologies have recently overshadowed the conventionally prevalent physical and chemical technologies (Chandran et al., 2020). In microbial technologies, microbes are used to clean up the contaminated environment and boost other allied technologies, such as phytoremediation and the breaking up of contaminants into small fractions using enzymes. The advantage of microbial technologies in the rehabilitation of contaminated environments is conferred by their complete nature, *in-situ* and *ex-situ* capability, ability to convert contaminants to their non-toxic form, shorter duration, cost-effectiveness, and eco-friendliness. Due to the many benefits of microbial technologies in decontaminating and rehabilitating contaminated environments, a Research Topic “Trends in Microbial Technologies in the Rehabilitation of Contaminated Environments” was proposed to explore the trends in this field. The topic aims to identify the most appropriate microbes for use in these technologies, as well as their diversity, methods, and molecular insights. This information will be used to target specific genes and traits that are relevant to the decontamination and rehabilitation of contaminated environments.

In microbial technologies, microbes are used to rehabilitate contaminated environments. The type of contaminants are heavy metals, pesticides, polycyclic aromatic hydrocarbon, oil, organic and inorganic chemical deposits, mine- and oil-explored wasteland, drilling, industrial effluents, municipal waste, dumping grounds, etc; plastics are the different major unwanted waste chemicals that contaminate the environment (Aktar et al., 2009; Machlis and Mcnutta, 2010; Othumpangat and Castranova, 2014; Wexler, 2014; Baruah et al., 2017; Briffa et al., 2020). The microbial technologies and basic parameters used to address the individual contaminants involved the assessment of microbial diversity, natural attenuation, biostimulation, bioaugmentation, and remediation. Investigations have reported the importance of parameters involved in bioremediation processes such as a microbial diversity assessment to understand the nature of microbes available in specific contaminated environments (Baruah et al., 2017), natural attenuation process (Kennedy, 2004), biostimulation (Seklemova et al., 2001), bioaugmentation (Garbisu et al., 2017), and overall microbe-assisted remediation approaches (Kumar et al., 2021). In addition, recent genes and traits of individual microbes were evaluated to reduce the targeted time of remediation (Bialy, 1997; Iyer et al., 2013; Puig et al., 2021). In the present Research Topic, five manuscripts were submitted for consideration, including a mini review on microbial enzymes for microplastic solution pesticide Diquat management through yeast (De Jesus and Alkendi), including insight into the genes (Wang et al.); arsenic and nitrogen contaminated groundwater treatment using *Hydrogenophega* sp. H7 (Fan et al.); and the treatment of oily sludge with a bacterial consortium (Hentati et al.). The diversified reports on microbes in the remediation of contaminated environments showed the importance of microbes in the rehabilitation, decontamination, or revival of contaminated environments.

## Trends in microbial investigations on the remediation of contaminated environments

From the above discussion, it is now the view that microbes play a vital role in recovering contaminated environments to their original state. Conceptualizing the use of microbes in the rehabilitation of a contaminated environment encompasses the diverse type of biochemical mechanisms that lead to a target's mineralization, transformation to a new product, and alteration of the state of the contaminants, e.g., metallic state. Conceptually, we observed that the use of microbial technology in the remediation of contaminants mostly involved removal, degradation, oxidation, and leaching, as well as groundwater. Overall, if we look at the type of contaminants in soil and water, mostly heavy metals, other minerals, organic pollutants, wastewater released from industries, municipal waste, and microbial technologies were mentioned in each subject. A Web of Science database search using the term “*bioremediation reports*” uncovered nearly 6,000 publications between 1988 and 2022, a significant increase in publications encompassing diverse fields.

**Figure 1:**
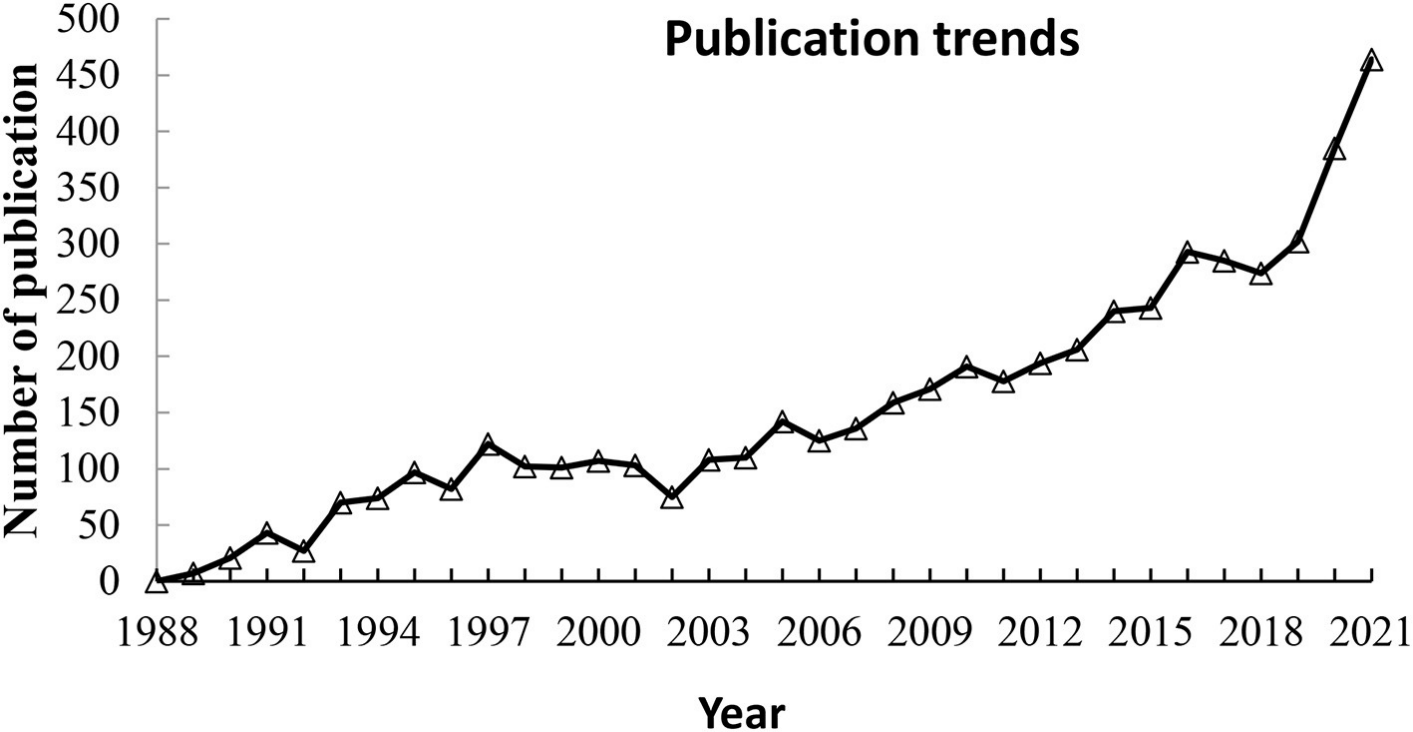
Publication evolution in the period 1989–2021 for the subject of bioremediation.

Analyses of the conceptual structure of the bioremediation of contaminated environment trends for different types of contaminated environments have mostly evaluated the overall diversity of microbial communities in specific environments and their role in decontamination. Based on the diversity of microbial community evaluation, researchers targeted the screening of bacteria most suited for degrading, mineralizing, or oxidizing contaminants, or ameliorating their toxic effects. The assessment of diverse microbial communities is reported to evaluate the entire environment, including water, soil, and industrial effluent. In evaluating microbes for remediation, many investigations also evaluated the toxicity of contaminants. The trends in microbial evaluation reported adsorption, biosorption, decolorization, reduction, biodegradation, and degradation. Based on the overall investigation, the appropriate bacteria were further screened for a microbial technology evaluation. In a microbial technology evaluation, both biotic and abiotic parameters were assessed for their full-proof technology development. In the latest trends in bioremediation, researchers try to identify the specific genes and traits responsible for decontamination. The latest overall strategy of research trends is the development of microbial full-proof technology (Figure 1).

It is evident that microbes play a vital role in the rehabilitation of contaminated environments through appropriate microbial technology and also have a diversified effect. To develop microbial technology in the remediation of contaminated environments, in most cases, the technology will be derivative of a case-specific program. To overcome the specific hurdles and to achieve universal acceptance, one must integrate the diverse parameters with pathway linking, the fate of the final product, etc. From a techno-economic viewpoint, including the total remediation of contaminants, the use of microbial technology has a long-lasting impact on the rehabilitation of the contaminated environment.

## Author contributions

HD: Writing—original draft, Writing—review and editing. PC: Writing—original draft, Writing—review and editing, Conceptualization. CA: Writing—original draft.

## References

[B1] Aktar W. Sengupta D. Chowdhury A. (2009). Impact of pesticides use in agriculture: their benefits and hazards. Interdiscip. Toxicol. 2, 1–12. 10.2478/v10102-009-0001-7 21217838 PMC2984095

[B2] Baruah R. Mishra S. K. Kalita D. J. Silla Y. Chauhan P. S. Singh A. K. Deka Boruah H. P. (2017). Assessment of bacterial diversity associated with crude oil-contaminated soil samples from Assam. Int. J. Environ. Sci. Technol. 14, 2155–2172. 10.1007/s13762-017-1294-2

[B3] Bialy H. (1997). Biotechnology, bioremediation, and blue genes. Neture Biotech. 15, 110. 10.1038/nbt0297-110 9035117

[B4] Briffa J. Sinagra E. Blundell R. (2020). Heavy metal pollution in the environment and their toxicological effects on humans. Heliyon 6, e04691. 10.1016/j.heliyon.2020.e04691 32964150 PMC7490536

[B5] Chandran H. Meena M. Sharma K. (2020). Microbial biodiversity and bioremediation assessment through omics approaches. Front. Environm. Chem. 1, 570326. 10.3389/fenvc.2020.570326

[B6] Garbisu C. Garaiyurrebaso O. Epelde L. Grohmann E. Alkorta I. P. (2017). lasmid-mediated bioaugmentation for the bioremediation of contaminated soils. Front. Microbiol. 8, 01966. 10.3389/fmicb.2017.01966 29062312 PMC5640721

[B7] Iyer R. Iken B. Damania A. A. (2013). Comparison of organophosphate degradation genes and bioremediation applications. Environ. Microb. Rep. 5, 787–798 10.1111/1758-2229.12095 24249287

[B8] Kennedy L. G. (2004). “Nine: Transportation and environmental justice,” in Running on Empty (Bristol: Policy Press). 10.51952/9781847426000.ch009

[B9] Kumar V. Roy S. Behera B. K. Swain H. S. Das B. K. (2021). Biofloc microbiome with bioremediation and health benefits. Front. Microbiol. 12, 741164. 10.3389/fmicb.2021.741164 34912305 PMC8667556

[B10] Machlis G. E. Mcnutta M. K. (2010). Scenario-building for the deepwater horizon Oil spill. Science. 329, 1018–1019. 10.1126/science.1195382 20798302

[B11] Othumpangat S. Castranova V. (2014). Oil spills. Encyclopedia Toxico. 2014, 677–681 10.1016/B978-0-12-386454-3.00359-6

[B12] Puig S. Jourdin L. Kalathil S. (2021). Editorial: Microbial Electrogenesis, Microbial Electrosynthesis, and Electro-bioremediation. *Front*. Microb. 12, 742479. 10.3389/fmicb.2021.742479 34589079 PMC8473817

[B13] Seklemova E. Pavlova A. Kovacheva K. (2001). Biostimulation-based bioremediation of diesel fuel: field demonstration. Biodegradation. 12, 311–316. 10.1023/A:1014356223118 11995824

[B14] Wexler P. (2014). Encyclopedia of Toxicology, US National Library of Medicine. Bethesda, MD, USA: Elsevier Inc.

